# Use of Novaluron-Based Autocidal Gravid Ovitraps to Control *Aedes* Dengue Vector Mosquitoes in the District of Gampaha, Sri Lanka

**DOI:** 10.1155/2020/9567019

**Published:** 2020-02-29

**Authors:** Gayan P. Withanage, Sameera D. Viswakula, Yasanthi Silva Gunawardene, Menaka D. Hapugoda

**Affiliations:** ^1^Molecular Medicine Unit, Faculty of Medicine, University of Kelaniya, Ragama, Sri Lanka; ^2^Department of Statistics, Faculty of Science, University of Colombo, Colombo 07, Sri Lanka

## Abstract

Dengue is the most important mosquito-borne viral infection in Sri Lanka causing an enormous social and economic burden in the country. In the absence of therapeutic drugs and the developed vaccines are under investigation, vector control is the best strategy to reduce the disease transmission. Therefore, the development of novel tools to control dengue vector mosquitoes has become the need of the hour. Novaluron is a recently developed Insect Growth Regulator (IGR) which inhibits chitin synthesis in immature stages of insects. The aim of the study was to identify the efficacy of a simple and cost-effective Autocidal Gravid Ovitrap (AGO) developed using Novaluron to control dengue outbreaks in the District of Gampaha, Sri Lanka. Laboratory and semifield experiments were performed to identify the activity range, optimum field dosage, and residual effects of Novaluron following the World Health Organization guidelines, and field experiments were performed in the Ragama Medical Officer of Health (MOH) area. Two study areas 800 m apart were selected and assigned as treated and control areas randomly. In each study area, 30 households were selected randomly. Each household was given two ovitraps, one placed indoors and the other placed outdoors. Mortality and survival counts were recorded separately for one-year time period and data were analyzed using a two-way repeated measures analysis of variance model. During the laboratory experiments, the adult emerging inhibition was 100% in all tested concentrations. The optimum field dosage was 2 ppm and the residual effect was 28 days. In the field experiments, significantly higher mortality counts were recorded in treated areas both indoor- and outdoor-placed AGOs. Two-factor repeated measures ANOVA followed by Tukey's test confirmed that the mean mortality count is high for the developed AGOs both indoor and outdoor settings. The developed AGO can be deployed to control both indoor and outdoor dengue vector mosquito populations, and in dengue-risk areas, the ovitrap will be supportive to local health authorities to enhance the efficiency of future vector control programs.

## 1. Introduction

Dengue is the most rapidly spreading mosquito-borne viral infection in the tropical and subtropical region of the world. Every year, more than 390 million dengue infections reported globally of which 96 million clinically manifest and approximately 3.9 billion people are living in these dengue-endemic countries. The causative agent of the infection is one of the four antigenically distinct serotypes of Dengue Virus (DENV) and the disease transmitted to humans mainly during the blood meal of *Aedes* (*Ae.*) mosquitoes predominantly by *Ae*. *aegypti* (Linnaeus), an urban vector who almost entirely feed on humans and breed on water-holding artificial containers, and the secondary vector is *Ae. albopictus* (Skuse). Rapid growth of human population, unplanned urbanization, scare sanitary facilities, and increased global travelling upsurge the transmission of the disease in endemic regions [1, 2]. In Sri Lanka, more than 30,000 dengue infections were reported every year making it a severe health and social burden in the country. The first dengue incidence was reported in Sri Lanka in 1962. Despite many efforts, in 2017, Sri Lanka experienced the most severe dengue outbreak with 186,101 infections island wide with over 250 dengue-related deaths [3]. Every year, nearly half of the incidences are reported from the Western Province which comprises Districts of Colombo, Gampaha, and Kalutara, and despite many efforts, the second highest number of dengue cases is reported from the District of Gampaha since 2010 [4–6]. Further, during the dengue epidemic in July 2017, the highest number of dengue cases was reported from the District of Gampaha [7]. Yet, there is neither licensed vaccine nor effective drug available; vector controlling through source reduction is the best strategy to control transmission of dengue in epidemic areas. The effectiveness of these programs is questionable mainly due to lack of resources and problems with timely application. Therefore, health authorities in Sri Lanka invest their attention recently towards development of novel tools for vector controlling programs as prime requirements.

**Figure 1:**
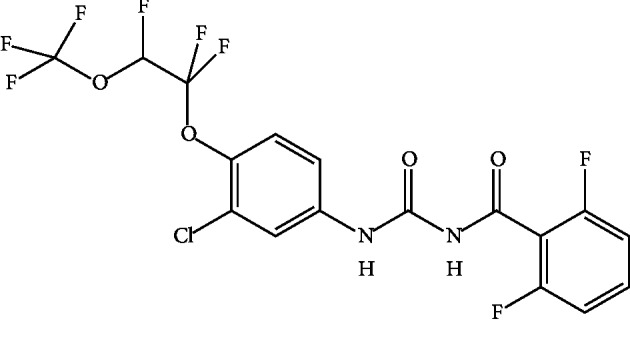
Structure of Novaluron (Source: National Center for Biotechnology Information. PubChem Database [16]).

Ovitrap is an artificial container that facilitates mosquitoes to lay eggs and used to monitor the population density of *Aedes* mosquitoes [8]. Lethal Autocidal Gravid Ovitraps (AGOs) can prevent trapped mosquito eggs proliferating into adult mosquitoes. These AGOs are dark containers facilitating female mosquitoes to lay eggs, but inside these containers, an insecticide is present at lethal dosage to mosquito larvae, an Insect Growth Regulator (IGR) or neurotransmitter inhibitor, which can be poisoned to larval stages. Therefore, pupae will not develop and this will lead to reduced mosquito population. This method is considered to be an effective method compared to the fumigation and other interior residual spraying programs because of reduced potential exposure of nontargets, residents, and applicators to the pesticide with little or no accumulation in the environment and minimum possibility to develop insecticide-resistant species, while creating potential cost savings [9, 10]. However, insecticide resistance of *Ae*. *aegypti* for organophosphates and pyrethroids has been reported previously [11, 12]. A recently developed IGR, Novaluron ((±)-1-[3-chloro-4-(1,1,2-trifluoro-2-trifluoro-methoxyethoxy)phenyl]-3-(2,6-difluorobenzoyl)urea) (Figure 1), a benzoylphenyl urea compound, is a chitin synthesis inhibitor affecting moulting stages of larval development after ingestion or contact triggering abnormal endocuticular deposition and abortive moulting [13, 14]. According to the Food and Agriculture Organization (FAO) specifications and evaluations, the solubility of Novaluron is 3 *μ*g/L at 20°C at neutral pH [13]. Experiments of Novaluron on toxicity levels for animals and drinking water demonstrate extensive metabolism of absorbed Novaluron and rapid excretion [15]. Therefore, Novaluron can be a better candidate as an active ingredient (AI) in AGO. Further, Novaluron is a commercially available IGR in Sri Lanka.

## 2. Materials and Methods

There is no AGOs available in the District of Gampaha yet to control dengue vector mosquito population in dengue high-risk areas. Therefore, this study is aimed at developing and identifying the efficacy of cost-effective and economical AGOs using Novaluron as the AI to reduce dengue vector mosquito population in Sri Lanka. In the study, Novaluron was subjected to both laboratory and field experiments to identify optimum concentrations and cost-effectiveness of the developed ovitraps. Technical material for the study was received as 100 g/L Emulsifiable Concentrate (EC) solution. Prior to initiating laboratory and field experiments, ethical permission for the experiment was obtained from the Ethical Review Committee of the Faculty of Medicine, University of Kelaniya, Sri Lanka (Ref. No. P/238/12/2014).

### 2.1. Laboratory Experiments to Identify Minimum Lethal Doses

Laboratory experiments were performed following guidelines of the World Health Organization (WHO) Pesticide Evaluation Scheme (WHOPES) [17] at the Molecular Medicine Unit, Faculty of Medicine, University of Kelaniya, Sri Lanka. Briefly, the solubility of Novaluron (100 g/L, Rimon 10 EC, Chemtura Corporation) was tested initially. Then, an aliquot of 20 mL stock solution at 10 ppm concentration was prepared using distilled water. Batches of 30 insectary-reared healthy and equally sized third instar larvae of *Ae. aegypti* and *Ae. albopictus* were exposed separately to a concentration gradient from 5.0 ppm to 0.01 ppm and the tap water control. Tap water was used to prepare the concentration gradient and mortality counts were recorded daily. Mosquito larvae were selected for controls for each test concentration following the same fashion and only tap water was used for the preparation of controls.

After the identification of the activity range of the Novaluron, a lower ranged concentration gradient was prepared from 3.0 ppm to 0.5 ppb. Small disposable 240 mL plastic cups with the height of 10 cm were used as test cups. The test cups were filled with aliquots of 200 mL solution of each concentration of the gradient. After the addition of solutions, the depth of the solution in the test cups was 7.5 cm. Batches of 30 (*n* = 30) insectary-reared healthy and equally sized third instar larvae of *Ae*. *aegypti* and *Ae*. *albopictus* were transferred separately to the test cups.

Five replicates were installed for each concentration and an equal number of controls were set up simultaneously with tap water for each species separately. During the experiments, mosquito larvae were provided with suspended larval foods at two-day intervals at a concentration of 10 mg/L and the tests were performed at room temperature with a photoperiod of 12 hours light followed by 12 hours dark (12L : 12D). All the test and control cups were covered with netting to prevent successfully emerged adults from escaping into the environment. Mortality was counted every other day until the larvae in treated test cups demised. The emerged adult mosquitoes in control test cups were demised by freezing [18]. The experiments were repeated three times for each assay.

### 2.2. Semifield Analysis to Identify Optimum Concentration and Design of AGOs

Semifield experiments were deployed in and around the Faculty of Medicine, University of Kelaniya, and monitored daily for a two-month time period (November-December, 2016), to identify efficacy in ecological settings, optimum field application dosage, and influence on abiotic parameter colour of AGO and falling debris.

#### 2.2.1. Identification of Optimum Field Dosage

Same test cups as used in the laboratory experiments were used for semifield experiments. Six concentrations, ranging from 3.0 ppm to 0.5 ppb, were tested to identify optimum field application dosage. Prepared test cups were filled with aliquots of 200 mL of water and kept 24-hour time period for conditioning prior to introduce a batch of 30 insectary-reared third instar larvae to each test cups. Small amount of larval foods were supplied as mentioned previously. After 3 hours of acclimation, aliquots of required volumes of Novaluron was added to each test cup and covered with nylon mesh. Five replicates were prepared from each concentration and the experiment was performed for *Ae*. *aegypti* and *Ae*. *albopictus* mosquito larvae separately. Mortality and survival counts were recorded in every seven days and placed traps were replenished with solution of experimenting concentration. The minimum concentration at which 100% mortality observed was considered the optimum field application dosage.

#### 2.2.2. Identification of Residual Effect

Another batch of late third instar larvae was introduced into each container to identify the residual effect of the selected concentration and larval food was added weekly. Larval survival was assessed every 48-hour time periods and without replenish test cups until no mortality observed or test cups were dried off.

#### 2.2.3. Identification of Effect of Colour and Falling Debris on the Developed AGOs

Both black- (*n* = 20) and white- (*n* = 20) coloured AGOs filled aliquots of 200 mL Novaluron test solution at 2 ppm concentration was placed randomly around the Faculty of Medicine and mortality counts were recorded weekly for two months. The demised mosquitoes were transferred to laboratory for species identification.

### 2.3. Field Studies to Identify Efficiency of the AGO

Following the identification of optimum concentration and effect of colour and abiotic debris on the developed AGOs, field studies were conducted.

#### 2.3.1. Site Selection

Ragama Medical Officer of Health (MOH) area, where one of the MOH areas with high population density in the District of Gampaha (Fig. [Supplementary-material supplementary-material-1]) and recently categorized as one of the dengue risk MOH areas after the massive dengue outbreak in 2017, was selected for the field studies. The study was conducted from 09 June 2017 to 08 June 2018 for a one-year time period. In the Ragama MOH area, two Grama Niladhari (GN) divisions, where similar number of dengue incidences were recorded and situated apart from 800 m, which exceed average flight distance of *Ae*. *aegypti* mosquitoes [19, 20], were selected and randomly assigned as treated and control areas [21].

#### 2.3.2. Preplacement Survey

Before placement of developed AGOs in study areas, an entomological survey was performed covering households and open areas in both study and control sites. All types of potential containers were inspected with minimum disturbances to environment as well as living status of the dwellers. During the entomological survey, total number of inspected containers, number of containers with water, and number of containers positive for larval or pupae stages were counted [21].

#### 2.3.3. Placement of AGOs

**Figure 2:**
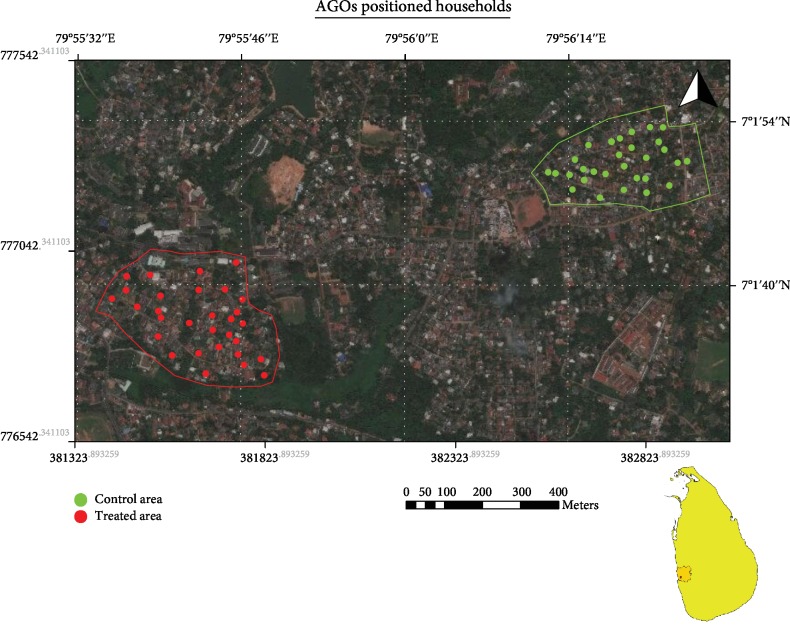
Selected households for the field experiments of the developed AGO.

In each study area, thirty households were selected from the middle of the each area for the study (Figure 2). An informed written consent was collected from all selected households prior to placement of the developed AGOs. Each selected household was given two developed AGOs, one was placed inside while the other was placed outside the premises [22]. The AGOs, filled with an aliquot of 200 mL of Novaluron solution at the effective concentration, were given to study areas' households while normal ovitraps filled with 200 mL of water were placed in the control site.

#### 2.3.4. Postplacement AGOs Survey

Placed ovitraps in the study and control sites were monitored weekly following the guidelines of the WHOPES [17]. Field study was conducted for one-year time period starting from June 2017. Dead and live mosquito larvae counts in each ovitrap were recorded separately for indoor and outdoor to evaluate the efficacy [23].

### 2.4. Analysis of Data

#### 2.4.1. Analysis of Laboratory and Semifield Data

At the end of the observation period in each study, the mortality and survival counts recorded from all replicates of each concentration were combined cumulatively and the effects of Novaluron is expressed in terms of adult emerging inhibition (IE %) which is the percentage of larvae that do not develop successfully into emerging adults, which is calculated following the formula:

(1) $$\begin{array}{c} \text{IE}\%=100-\left(\frac{T\times 100}{C}\right), \end{array}$$

where *T* is the percentage emergence in treated batches and *C* is the percentage emergence in control batches. The probit regression analysis was performed using the IE values obtained at each concentration to determine IE_50_ and IE_90_ values using Minitab 17 statistical software at 5% significance level [17].

#### 2.4.2. Analysis of Field Data

Statistical analyses were performed at 95% confidence level, unless stated otherwise. The recorded mortality counts of the field experiments were analyzed using a two-way repeated measure ANOVA model [24, 25]. The treatment group (control versus test) and the position placed (indoor versus outdoor) were considered as fixed effects, while the week is treated as the repeated measure. The model was built incorporating main effects and interactions between the treatment group and the AGOs' placed position in households. Then, Tukey's multiple comparison was conducted at 5% level to identify the significant differences of the mean effects [25, 26].

## 3. Results

### 3.1. Laboratory Studies on Designing the Ovitrap

**Figure 3:**
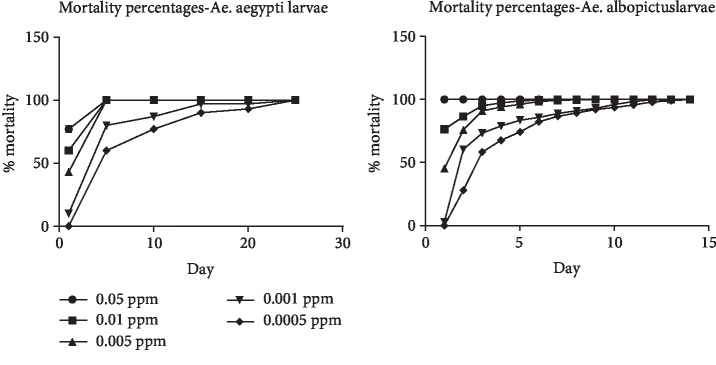
Mortality percentage variations over the experiment time period for both *Ae*. *aegypti* and *Ae*. *albopictus* mosquito larvae.

During the initial concentration gradient bioassay, all the mosquito larvae in both *Ae*. *aegypti* and *Ae*. *albopictus* were dead in the first day of the experiment. In the lower ranged concentration gradient bioassay, in *Ae. albopictus*, mosquito larvae test cups with lowest concentration (0.5 ppb) were demised after 14 days and 100% mortality was observed on the 25^th^ day for *Ae. aegypti* (Figure 3). The mortality counts recorded after two weeks were subjected to probit regression analysis and dosage-response results indicated 50% Lethal Concentrations (LD_50_) and LD_90_ for *Ae*. *albopictus* were 0.3 ppb and 0.4 ppb, and for *Ae*. *aegypti*, 0.2 ppb and 1.0 ppb, respectively.

### 3.2. Semifield Experiment Findings

In the semifield experiments, tests were initially conducted to identify the optimum field dosage of Novaluron. In the experiments on susceptibility of *Ae. aegypti*, after 21 days, pupae (*n* = 34) were observed in 0.5 ppb concentration test cups, and after 28 days, pupae (*n* = 8) were observed in 1 ppb concentration test cups. Further, one larvae (*n* = 1) in 0.5 ppm test cups were pupated after 28 days and in 0.5 ppb concentration test cups, 50.6% (*n* = 76) of larvae were pupated. In the experiments for *Ae*. *albopictus*, 100% mortality was observed in both 3 and 2 ppm test concentrations on the 7^th^ day, and by the 14^th^ day, all larvae were demised in all replicates in 1 ppm test concentration. Similarly, to *Ae. aegypti*, the test larvae were pupated (*n* = 5) after 21-day time period in 0.5 ppb concentration test cups during the experiment on *Ae. albopictus* larvae. After 28 days, one larvae (*n* = 1) was pupated in 1.0 ppb concentration, and in 0.5 ppb concentration test cups, 18.7% (*n* = 28) of larvae were pupated.

During the identification of residual effects of Novaluron, bioassays were performed for *Ae*. *aegypti* and *Ae*. *albopictus* separately. In the test, 94.7%, 81.3%, and 66.3% of *Ae*. *aegypti* larvae were demised after 7-day observation periods at 3, 2, and 1 ppm concentration test cups, respectively. In the assay, 100% mortality was observed in the 14^th^ day in 3 ppm concentration in all replicates and another batch of larvae were introduced. In the 21^st^ day, 100% mortality was observed in test cups of 2 ppm concentration and another batch is introduced. Same day in the 1 ppm concentration, only 92% mortality was observed. In the 27^th^ day, 100% mortality was observed in 3 and 2 ppm replicates while only 99.3% mortality was observed in the 1 ppm test cups. During the residual effect bioassay on *Ae. albopictus*, 100% mortality was observed in both 3 and 2 ppm concentration replicates after 7^th^ days and another batch was introduced to the test cups. Nevertheless, 100% mortality was observed in the replicates on 1 ppm concentration on the 14^th^ day and another batch was introduced. In the 3 and 2 ppm test replicates, the secondly introduced batch was demised completely on the 21^st^ day of observation and another batch was introduced into the test replicates. Meantime, 83.3% mortality was observed on the 21^st^ day in the 1 ppm test concentrations. On the 27^th^ day observation, 99.3% and 85.3% mortality was observed with the thirdly introduced batch in 3 and 2 ppm replicates, respectively, while 98.7% mortality was observed with secondly introduced batch in 1 ppm test replicates. On the 28^th^ day of the observation period, all the test cups were dried out and bioassays were discontinued and none of the larvae had pupated during the experiment period. Since only 99.3% mortality was observed in 1 ppm test concentration residual effect identification bioassay in *Ae*. *aegypti*, the optimum concentration for large-scale field investigation was selected as 2 ppm and residual effect was 28 days.

During the identification of effects of colour and falling debris in developed AGOs, on the 14^th^ day, demised first instar mosquito larvae (*n* = 37) were observed in the black-coloured AGOs which were placed with 200 mL aliquots of 2 ppm Novaluron solution, however, without introducing any mosquito larvae. During the 2 months of observation period, none of the white-coloured AGOs were positive for mosquito larvae. Laboratory identification of the dead larvae in these traps confirmed that all the dead mosquito larvae belong to genus *Aedes*.

### 3.3. Field Trials of the Developed AGOs

**Figure 4:**
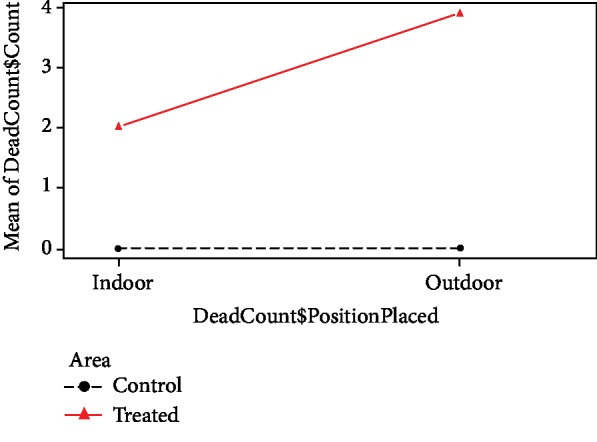
Interaction plot of the ANOVA model.

Field experiments were conducted in the Ragama MOH area and the treated AGOs were placed in Rampitiya GN division and control ovitraps were deployed in Dambuwa GN division. These GN divisions are apart from 810 m each other and share similar population densities and distribution of dengue incidences. Before the placement of ovitraps in both GN divisions, an entomological survey was performed. During the entomological survey, same number of wet containers (*n* = 19) were recorded from both study areas. However, in both study areas, none of the wet containers were positive for immature stages of *Aedes* dengue vector mosquitoes and no adult dengue vector mosquito was found during the entomological survey. In the field trial, stock solutions were prepared at 10^4^ ppm concentration and therefore, aliquots of 40 *μ*L of Novaluron was added to each field testing test cup to reach the 2 ppm test concentration. In the postplacement survey, mortality and survival counts of mosquito larvae were recorded separately for each indoor- and outdoor-placed ovitrap separately and the collected data were analyzed using a two-way repeated measures ANOVA to evaluate the main and interaction effects of mortality counts in treated and control areas in the study (Table 1). The interaction treatment group∗position placed was significant at 5% level confirming that the effect of treatment group on mean mortality count is different for different levels of position placed. This implied that the effect of AGO is different in indoors and outdoors. The Tukey's multiple comparison showed different mean effects except for control in and control out combinations (Table 2). From the profile plots (Figure 4), it can be seen that the mean death count increases for treatment, when moving from inside to outside.

## 4. Discussion

Novaluron has initially been used as a pest controller in food agriculture industries. Even though the exact mode of action is still not clear, its capability to inhibit exoskeleton formation in immature stages of insects has enabled it to be used for a broad range of applications. The Novaluron 10 EC solution dissolves well in water, and therefore, tap water was used to prepare a dilution series for laboratory and field experiments with the objective of minimizing the costs and improving convenience and feasibility for end users [27, 28]. However, stock solutions were prepared using distilled water following the WHO guidelines [17].

More attention was paid when selecting mosquito larvae for the laboratory and semifield experiments to ensure they were healthy and in the same stage and size. During the laboratory experiments, no mortality was observed in controls and all the larvae were pupated and emerged into adult mosquitoes, and therefore, mortality counts of treated test cups were directly taken into analysis without Abbott's correction [29]. The maximum residual limit, established by the Codex Alimentarius Commission in United States, of Novaluron for pone-type fruits was 3 ppm [30]. Previous study conducted on autodissemination strategy of IGR on mosquitoes stated that the limit of detection of Novaluron was 0.5 ppb [28]. Therefore, the experiments were performed in the range of 3.0 ppm to 0.5 ppb in the study. During the lower ranged concentration bioassay, *Ae*. *aegypti* survived more days than *Ae*. *albopictus* even though they died eventually. This may be attributed to higher tolerance to insecticides and environmental changes reported in *Ae*. *aegypti* due to *kdr* and other mutations [31, 32]. Further, the IE% was 100% for all the test concentrations for both the species and the LD_50_ and LD_90_ values for *Ae. aegypti* were higher than *Ae*. *albopictus*. Laboratory experiments were discontinued at 0.5 ppb test concentration as previous literatures mentioned no effect of Novaluron on survival of mosquito larvae beyond 0.1 ppb [33].

Mosquito larvae of the two species behaved differently in semifield trials when compared with laboratory bioassays. Even though no pupae were observed in laboratory experiments in 0.5 ppb concentration, 50.6% of the *Ae*. *aegypti* larvae and 18.7% of *Ae. albopictus* larvae were pupated at the end of semifield experiments. Moreover, pupa were observed in 1.0 ppb concentration in both species and 1.3% of *Ae. aegypti* larvae were pupated in 0.5 ppm test cups while all the larvae in *Ae. albopictus* were dead. Semifield experiments on investigating optimum field dosages were discontinued once the pupae were observed and tests were not conducted until adult emergence to minimize the possibility of developing resistance strains. During the identification of residual effects of Novaluron at studied concentrations, only 99.3% of *Ae. aegypti* larvae were demised at 1 ppm test cups prior to one day before all test cups dried. Therefore, 2 ppm concentration was selected for field experiments and the different observations during semifield experiments may be due to more exposure to natural environment conditions. Furthermore, the observed concentrations in the current study are higher than previous study conducted in Mexico [34] and it may be due to geographical variations among *Aedes* dengue vector species [35].

In the field experiments, the areas were selected apart from 800 m which is more than the flight range of dengue vector mosquitoes [19]. During the preplacement entomological survey, no breeding containers for *Aedes* dengue vector mosquitoes were found and it may be due to high atmospheric temperature and humidity with low rainfalls during the month of April in Sri Lanka [36]. During the placement, one ovitrap placed indoor and one placed outdoor to increase the oviposition rates as mentioned in previous studies and the placed ovitraps were monitored weekly to ensure no pupae development on the test concentration under uncontrolled environment conditions.

Analysis of indoor and outdoor mortality counts in treated and control areas indicated that the mortality counts were significantly higher in the treated area, where AGOs were placed, compared to the control area. Further, the dead counts in treated indoor AGOs were higher than control indoor-placed ovitraps suggesting that the developed ovitraps can be used to control resting dengue vector mosquito populations in inside households in dengue endemic areas. Interestingly, as the result of the interaction plot, even higher and increasing death counts were observed when moving from indoors to outdoors in the treated area. Such a difference was not observed in control area. The observation indicates that the developed AGOs can also efficiently reduce outdoor dengue vector mosquito population. Further, no animal poisoning of Novaluron in the AGOs was reported in the treated area during the field experiments.

**Figure 5:**
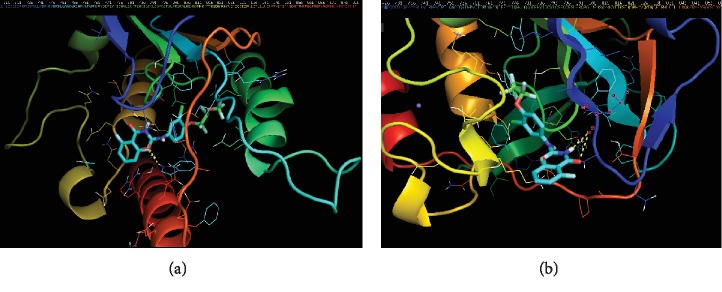
Binding of Novaluron to chitin synthase. (a) Binding of Novaluron to model 1 of chitin synthase. Novaluron interacts to Trp-872 with 2.6 Å bond length. (b) Binding of Novaluron to model 2 of chitin synthase. Novaluron interacts to Leu-726 with 2.6 and 3.3 Å bond lengths.

The key enzyme of chitin synthesis of insects is chitin synthase [37]. The exact mode of mechanism of Novaluron is not crystal clear even though it is predicted to be a chitin synthesis inhibitor. There are number of sequences available for insect chitin synthesis in UniProt; however, no crystal structure is available yet in the Protein Data Bank (PDB). Therefore, three-dimensional structures were generated with available chitin synthase sequence of *Ae*. *aegypti* [38] using SWISS-MODEL [39]. Results of the molecular docking experiments performed in AutoDock software [40, 41] indicated that binding affinity of Novaluron to model 1 of chitin synthase is -9.1 kcal/mol and Novaluron interacts with tryptophan (try) residue at 872 position. Further, binding affinity for the best interaction model developed for Novaluron and model 2 of chitin synthase was -8.5 kcal/mol and Novaluron interacts with leucine (leu) residue at 726 position (Figure 5). These results indicated that Novaluron interacts with mosquito chitin synthase with stable interaction. However, further experiments will be needed to characterize the activity of Novaluron.

Novaluron is a novel IGR and its efficiency can be led to many successful applications due to its noncarcinogenic nature [15] and no mosquito resistant have been reported yet to the best of our knowledge. However, previous studies have mentioned the susceptibility of Novaluron to Ultraviolet (UV) rays in sunlight and organic pollutants [42]. Therefore, these limitations need to be addressed near future. On the other hand, this is indicative that environmental pollution with Novaluron may not be long lasting making it a favorable mosquito control agent.

## 5. Conclusions

In Sri Lanka, controlling of indoor dengue vector population in dry seasons is a problem to health authorities and the developed AGO in the present study will be a great solution to reduce the impact of future outbreak, disease transmission, healthcare burdens, and possible mortalities. Further, due to the simplicity of the developed AGO and easiness for end users, the ovitrap can be distributed by local health authorities in dengue-risk areas to control disease transmission. An integrated approach of deployment of the AGO with vector control programs will increase the efficiency of future disease-controlling programs. Further experiments need to be conducted to overcome the potential limitation of Novaluron.

## Figures and Tables

**Table 1:** ANOVA model evaluation of the efficacy of the developed AGO in the study areas.

Variable	Df	Sum Sq	Mean Sq	*F* value
Treatment group	1	11276	11276	622.03^∗^
Position placed	1	913	913	50.37^∗^
Treatment group∗position placed	1	911	911	50.25^∗^

^∗^Significantly different interactions during multiple comparisons of means for each study group. *p* values at 95% family-wise confidence level were considered significant differences. Df: degree of freedom; Sum Sq: sum of squares; Mean Sq: mean squares.

**Table 2:** Tukey's multiple comparisons of means.

Interaction type	Difference in mean level
Treated:In-Control:In	2.006^∗^
Treated:Out-Control:In	3.882^∗^
Control:Out-Treated:In	-2.006^∗^
Treated:Out-Treated:In	1.876^∗^
Treated:Out-Control:Out	3.881^∗^
Control:Out-Control:In	0.001

^∗^Significantly different interactions during multiple comparisons of means for each study group. *p* values at 95% family-wise confidence level were considered significant differences. In: indoor; out: outdoor.

## Data Availability

The number of dengue incidences available in the MOH office, Ragama. Furthermore, data on mortality counts used to support the findings are available from the corresponding author upon request.

## Abbreviations

*Ae.*: *Aedes*
AI: Active ingredient
AGO: Autocidal gravid ovitraps
ANOVA: Analysis of variance
MOH: Medical officer of health
PHI: Public health inspector
UV: Ultraviolet
WHO: World Health Organization
WHOPES: WHO pesticide evaluation scheme.

## Supplementary Materials

Supplementary Materials Figure S1: population density and distribution of dengue incidences in the District of Gampaha.

## References

[1] Gubler D. J. (1998). Dengue and dengue hemorrhagic fever. *Clinical Microbiology Reviews*.

[2] Bhatt S., Gething P. W., Brady O. J. (2013). The global distribution and burden of dengue. *Nature*.

[3] Organization, WH (2017). Dengue fever – Sri Lanka. https://www.who.int/csr/don/19-july-2017-dengue-sri-lanka/en/.

[4] Withanage G. P., Viswakula S. D., Nilmini Silva Gunawardena Y. I., Hapugoda M. D. (2018). A forecasting model for dengue incidence in the District of Gampaha, Sri Lanka. *Parasites & Vectors*.

[5] Arunachalam N., Tana S., Espino F. (2010). Eco-bio-social determinants of dengue vector breeding: a multicountry study in urban and periurban Asia. *Bulletin of the World Health Organization*.

[6] Abeyewickreme W., Wickremasinghe A. R., Karunatilake K., Sommerfeld J., Axel K. (2012). Community mobilization and household level waste management for dengue vector control in Gampaha district of Sri Lanka; an intervention study. *Pathogens and Global Health*.

[7] Epidemiology Unit SL Distribution of notification(H399) dengue cases by month. http://www.epid.gov.lk/web/index.php?option=com_casesanddeaths&Itemid=448&lang=en.

[8] Sivagnaname N., Gunasekaran K. (2012). Need for an efficient adult trap for the surveillance of dengue vectors. *The Indian Journal of Medical Research*.

[9] Mackay A. J., Amador M., Barrera R. (2013). An improved autocidal gravid ovitrap for the control and surveillance of Aedes aegypti. *Parasites & Vectors*.

[10] Health, VDo (2016). *Zika virus disease - response annex*.

[11] Ponlawat A., Scott J. G., Harrington L. C. (2005). Insecticide susceptibility of Aedes aegypti and Aedes albopictus across Thailand. *Journal of Medical Entomology*.

[12] McAllister J. C., Godsey M. S., Scott M. L. (2012). Pyrethroid resistance in Aedes aegypti and Aedes albopictus from Port-au-Prince, Haiti. *Journal of Vector Ecology*.

[13] World Health Organization (2005). *Pesticide Residues in Food - 2005: Evaluations. Part I, Part I. in Joint meeting of the FAO panel of experts on pesticide residues in food and environment*.

[14] Djeghader N. E. H., Djeghader N. E., Aïssaoui L., Amira K., Boudjelida H. (2014). Impact of a chitin synthesis inhibitor, novaluron, on the development and the reproductive performance of mosquito Culex pipiens. *World Applied Sciences*.

[15] World Health Organization (2007). *Novaluron in Drinking-water: Use for Vector Control in Drinking-water Sources and Containers*.

[16] Database, NCfBIP Novaluron, CID=93541. https://pubchem.ncbi.nlm.nih.gov/compound/Novaluron.

[17] Zaim M., WHOPES, World Health Organization (2005). *Guidelines for Laboratory and Field Testing of Mosquito Larvicides*.

[18] Ljungström I., Moll K., Perlmann H., Scherf A., Wahlgren M. (2008). *Methods in Malaria Research*.

[19] Honório N. A., Silva Wda C., Leite P. J., Gonçalves J. M., Lounibos L. P., Lourenço-de-Oliveira R. (2003). Dispersal of *Aedes aegypti* and *Aedes albopictus* (Diptera: Culicidae) in an urban endemic dengue area in the State of Rio de Janeiro, Brazil. *Memórias do Instituto Oswaldo Cruz*.

[20] World Health Organization Dengue control - the mosquito. http://www.who.int/denguecontrol/mosquito/en/.

[21] Sithiprasasna R., Mahapibul P., Noigamol C. (2003). Field evaluation of a lethal ovitrap for the control of *Aedes aegypti* (Diptera: Culicidae) in Thailand. *Journal of Medical Entomology*.

[22] Lourenço-de-Oliveira R., Lima J. B. P., Peres R., da Costa Alves F., Eiras Á. E., Codeço C. T. (2008). Comparison of different uses of adult traps and ovitraps for assessing dengue vector infestation in endemic areas. *Journal of the American Mosquito Control Association*.

[23] Rohani A., Suzilah I., Malinda M. (2011). Aedes larval population dynamics and risk for dengue epidemics in Malaysia. *Tropical Biomedicine*.

[24] Davis C. S. (2002). Statistical methods for the analysis of repeated measurements. *International Journal of Epidemiology*.

[25] Field A. M. (2013). *Discovering Statistics Using R*.

[26] McHugh M. L. (2011). Multiple comparison analysis testing in ANOVA. *Biochemia Medica*.

[27] Khan G. Z., Khan I., Khan I. A., Alamzeb, Salman M., Ullah K. (2016). Evaluation of different formulations of IGRs against *Aedes albopictus* and *Culex quinquefasciatus* (Diptera: Culicidae). *Asian Pacific Journal of Tropical Biomedicine*.

[28] Swale D. R., Li Z., Kraft J. Z. (2018). Development of an autodissemination strategy for the deployment of novel control agents targeting the common malaria mosquito, Anopheles quadrimaculatus say (Diptera: Culicidae). *PLoS Neglected Tropical Diseases*.

[29] Abbott W. S. (1925). A method of computing the effectiveness of an insecticide. *Journal of Economic Entomology*.

[30] Federal Register U. Novaluron; Pesticide Tolerances. Rules and Regulations 2015. https://www.federalregister.gov/documents/2015/07/22/2015-17676/novaluron-pesticide-tolerances.

[31] Dickens B. L., Sun H., Jit M., Cook A. R., Carrasco L. R. (2018). Determining environmental and anthropogenic factors which explain the global distribution ofAedes aegyptiandAe. albopictus. *BMJ Global Health*.

[32] Amelia-Yap Z. H., Chen C. D., Sofian-Azirun M., Low V. L. (2018). Pyrethroid resistance in the dengue vector Aedes aegypti in Southeast Asia: present situation and prospects for management. *Parasites & Vectors*.

[33] Su T., Mulla M. S., Zaim M. (2003). Laboratory and field evaluations of novaluron, a new insect growth regulator (IGR), against Culex mosquitoes. *Journal of the American Mosquito Control Association*.

[34] Arredondo-Jiménez J. I., Valdez-Delgado K. M. (2006). Effect of Novaluron (Rimon®10 EC) on the mosquitoes *Anopheles albimanus, Anopheles pseudopunctipennis, Aedes aegypti, Aedes albopictus and Culex quinquefasciatus* from Chiapas, Mexico. *Medical and Veterinary Entomology*.

[35] de Lourdes Macoris M., Martins A. J., Andrighetti M. T. M., Lima J. B. P., Valle D. (2018). Pyrethroid resistance persists after ten years without usage against Aedes aegypti in governmental campaigns: lessons from São Paulo State, Brazil. *PLOS Neglected Tropical Diseases*.

[36] Studies, CfcC Climate of Sri Lanka. http://www.meteo.gov.lk/index.php?option=com_content&view=article&id=94&Itemid=310&lang=en.

[37] Merzendorfer H. (2011). The cellular basis of chitin synthesis in fungi and insects: common principles and differences. *European Journal of Cell Biology*.

[38] Ibrahim G. H., Smartt C. T., Kiley L. M., Christensen B. M. (2000). Cloning and characterization of a chitin synthase cDNA from the mosquito Aedes aegypti. *Insect Biochemistry and Molecular Biology*.

[39] Waterhouse A., Bertoni M., Bienert S. (2018). SWISS-MODEL: homology modelling of protein structures and complexes. *Nucleic Acids Research*.

[40] Trott O., Olson A. J. (2010). AutoDock Vina: improving the speed and accuracy of docking with a new scoring function, efficient optimization, and multithreading. *Journal of Computational Chemistry*.

[41] Ballante F., Marshall G. R. (2016). An automated strategy for binding-pose selection and docking assessment in structure-based drug design. *Journal of Chemical Information and Modeling*.

[42] World Health Organization (2004). *Report of the Eighth WHOPES Working Group Meeting*.

