# Supplementary figures and images for: Use of Novaluron-Based Autocidal Gravid Ovitraps to Control Aedes Dengue Vector Mosquitoes in the District of Gampaha, Sri Lanka
Source: Biomed Res Int. 2020 Feb 29;2020:9567019. doi: 10.1155/2020/9567019 (PMC7071800; doi:10.1155/2020/9567019)

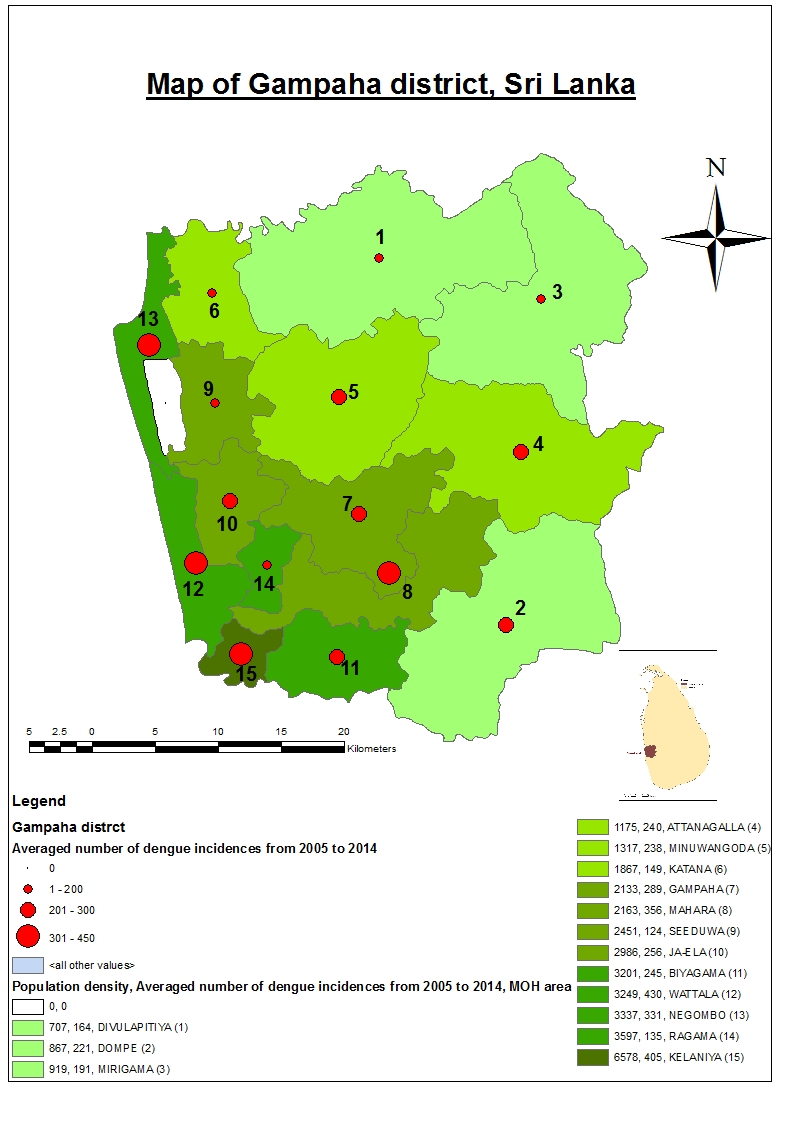

Supplement: Supplementary Materials — Figure S1: population density and distribution of dengue incidences in the District of Gampaha. [file 9567019.f1.tif]
